# Adherence to home nutritional management in patients following esophagectomy: a qualitative study based on the Health Belief Model

**DOI:** 10.3389/fnut.2026.1878338

**Published:** 2026-07-17

**Authors:** Qian Ding, Shuang He, Shuang Quan, Yan-yan Ning, Feng-juan Yang, Wenqiang Zhang

**Affiliations:** 1 Transformation Engineering Research Center, Henan Provincial Key Medicine Laboratory of Nursing, Henan Provincial People’s Hospital; Zhengzhou University People’s Hospital, Henan University People’s Hospital, Zhengzhou, Henan, China; 2 School of Nursing and Health, Zhengzhou University, Zhengzhou, Henan, China

**Keywords:** esophageal cancer, Health Belief Model, home-based nutritional management, influencing factors, nutritional adherence, qualitative research, surgery

## Abstract

**Objective:**

To identify factors influencing adherence to home-based nutritional management in post-esophagectomy patients using the Health Belief Model (HBM), thereby addressing the current gap in understanding behavioral drivers and guiding the development of theory-based transitional nutritional interventions.

**Methods:**

Guided by the HBM, we conducted a qualitative descriptive study. Using purposive sampling, semi-structured interviews were conducted with 18 patients who had undergone radical esophagectomy at a tertiary hospital in Henan Province, China, between October and December 2025. Data were analyzed using directed content analysis. The study was approved by the hospital ethics committee [Approval No. (2025)154].

**Results:**

The barriers to adherence included insufficient perception of malnutrition threats (lack of awareness of long-term risks and cognitive biases), excessive perceived barriers (symptom-related eating difficulties, financial burden, negative emotions, and lack of nutrition knowledge and skills), low self-efficacy, and lack of effective cues to action. The factors facilitating adherence (termed facilitators) included a clear perception of postoperative malnutrition risk, explicit perception of the benefits of the nutritional regimen, strong self-efficacy (active learning and strategy development, positive feedback loop from experience accumulation to confidence building, and internalization of nutritional management), and positive internal and external cues to action (sensitization to physical signals and self-monitoring, and synergistic effects of multi-source support networks).

**Conclusion:**

Adherence to home-based nutritional management after esophageal cancer surgery is influenced by multiple factors. Healthcare providers should pay close attention to patients’ experiences, identify and mitigate barriers, and reinforce facilitators. Interventions such as risk communication, skill training, and self-efficacy enhancement can improve adherence, nutritional status, and quality of life.

## 1 Introduction

Esophageal cancer is one of the most common malignant tumors of the digestive system; it imposes a substantial disease burden worldwide (1). China bears the heaviest burden globally. In 2022, the incidence and mortality rates of esophageal cancer in China were 15.87 per 100,000 and 13.28 per 100,000, respectively (2). Surgery is currently the first-line treatment for esophageal cancer. However, due to surgical trauma and intraoperative digestive tract reconstruction, the incidence of malnutrition after esophagectomy is as high as 60–80% (3, 4). Standardized nutritional management can help reduce the incidence of postoperative malnutrition and improve patients’ clinical outcomes and quality of life (5).

**Figure 1:**
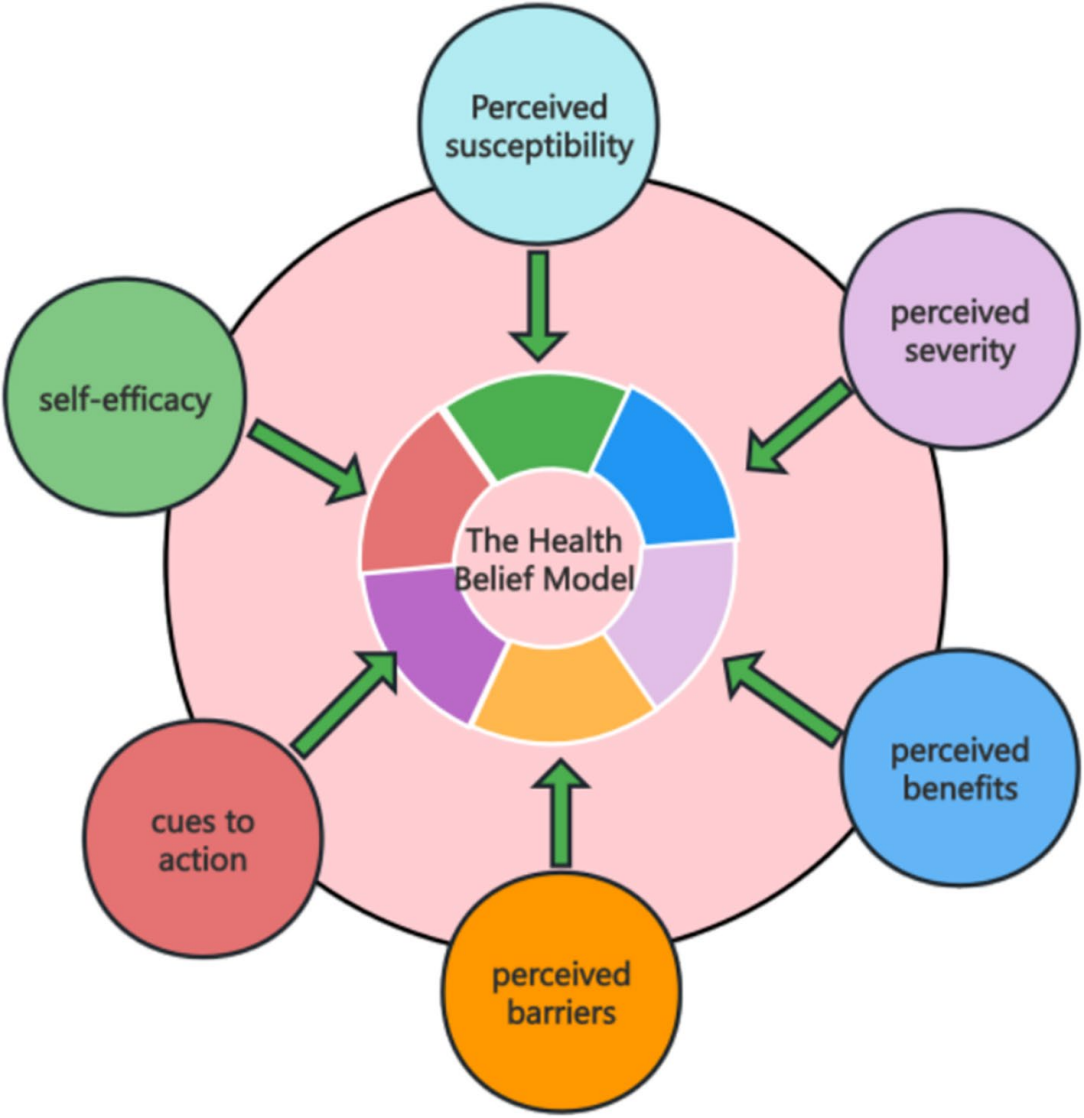
Theoretical framework.

However, existing studies have mostly focused on nutritional status surveys or the development of nutritional interventions for patients who have undergone surgery for esophageal cancer, with insufficient attention paid to the actual nutritional practices patients follow at home. Few studies have adopted a behavioral science perspective to explore the psychological and social factors that influence patients and their families to adhere to nutritional regimens voluntarily and consistently in home settings (6, 7). Instituting changes in behavior is a complex process. The Health Belief Model (HBM) is an important theoretical framework for explaining and predicting health behaviors (8). The HBM posits that individuals’ health behaviors are influenced by a combination of the following factors: perceived susceptibility, perceived severity, perceived benefits, perceived barriers, cues to action, and self-efficacy (Figure 1) (9). In recent years, the HBM has been widely applied in chronic disease management and behavioral interventions for patients with cancer (10–12).

Based on this, the present study used the HBM as a theoretical framework and adopted a qualitative approach to explore in depth the barriers to and facilitators of adherence to home-based nutritional management among patients after esophageal cancer surgery. The aim of this study was to fill the current gap in understanding behavioral drivers and provide a reference for constructing more targeted and feasible transitional nutritional nursing interventions.

## 2 Methods

### 2.1 Participants

Purposive sampling was used to recruit patients who had undergone radical esophagectomy and had been treated at the thoracic surgery department of a tertiary hospital in Henan Province, between October and December 2025.

The inclusion criteria were: pathologically confirmed esophageal cancer; home rehabilitation for ≥1 month after surgery; good comprehension and verbal communication skills; aware of their diagnosis and willing to participate voluntarily.

The exclusion criteria were: mental illness or other severe cardiac, cerebral, or pulmonary complications and withdrawal during the study. The sample size was determined based on the information saturation. Finally, 18 patients were enrolled to participate in the interviews. The study was approved by the hospital ethics committee [Approval No. (2025)154].

### 2.2 Methods

#### 2.2.1 Interview guide

The interview guide was developed based on the HBM, a literature review, and team brainstorming. Two patients were pre-interviewed and the guide was revised accordingly. The final interview questions were:

1. After discharge, what have you done regarding home-based diet and nutritional management?
2. During your recovery at home, what specific benefits has “eating well and managing nutrition well” brought to your rehabilitation?
3. What consequences do you think might occur if you do not follow the nutritional guidance provided by healthcare professionals?
4. How confident are you in following the nutritional guidance long-term? What things make you feel “I can do it,” and what things make you feel “I might not be able to”?
5. Who has helped you implement the nutritional plan after discharge? What other help would you like to receive?

#### 2.2.2 Data collection

Semi-structured face-to-face interviews were conducted to collect the data. Before each interview, the purpose, methods, content, and confidentiality principles were explained and written informed consent was obtained. The interviews were audio recorded with the patient’s permission. A relaxed atmosphere was maintained to encourage free expression. The interviewer avoided leading questions, noted keywords and nonverbal cues (expressions, tone, and gestures), and used appropriate probes and summaries. Each interview lasted 30–40 min. Reflective journals were written immediately after each interview and the transcripts were returned to the participants for confirmation.

#### 2.2.3 Data analysis

All audio recordings were transcribed verbatim into written text by two independent researchers within 24 h of each interview. Data were analyzed via Colaizzi’s seven-step phenomenological approach (13, 14), detailed as follows: (I) The principal investigator and a postgraduate student trained in qualitative methodologies read transcripts repeatedly and thoroughly to grasp an overall understanding of the data; (II) Two researchers separately extracted statements related to adherence to home-based nutritional management and identified meaningful sentences and paragraphs as units of analysis; (III) Each researcher conducted open coding for every unit of analysis to label recurrent, meaningful concepts; (IV) A categorization framework was built based on the five core constructs of HBM Researchers then individually assigned generated codes to corresponding categories; (V) Similar codes were grouped, classified and abstracted to develop preliminary themes and subthemes; (VI) Definitions and descriptions were formulated for all preliminary themes and subthemes, with typical participant quotations selected as supporting illustrations; (VII) The emergent findings were sent back to participants for member checking to ensure alignment with their original experiences. Member checking to ensure alignment with their original experiences.

Throughout coding and categorization, the research team predetermined the five core HBM constructs as the initial coding framework to guide thematic classification. The two coders met twice weekly to cross-check codes and evaluate coding consistency. Where discrepancies in code allocation emerged, each researcher elaborated their coding logic, and consensus was reached through group discussion. If disagreements could not be resolved, a third qualitative research specialist was consulted to mediate the conflict. Final agreement was achieved via team deliberation to guarantee the trustworthiness and consistency of analytical outcomes. Additionally, this study was reported in accordance with the Consolidated Criteria for Reporting Qualitative Research (COREQ) (15).

## 3 Results

**Figure 2:**
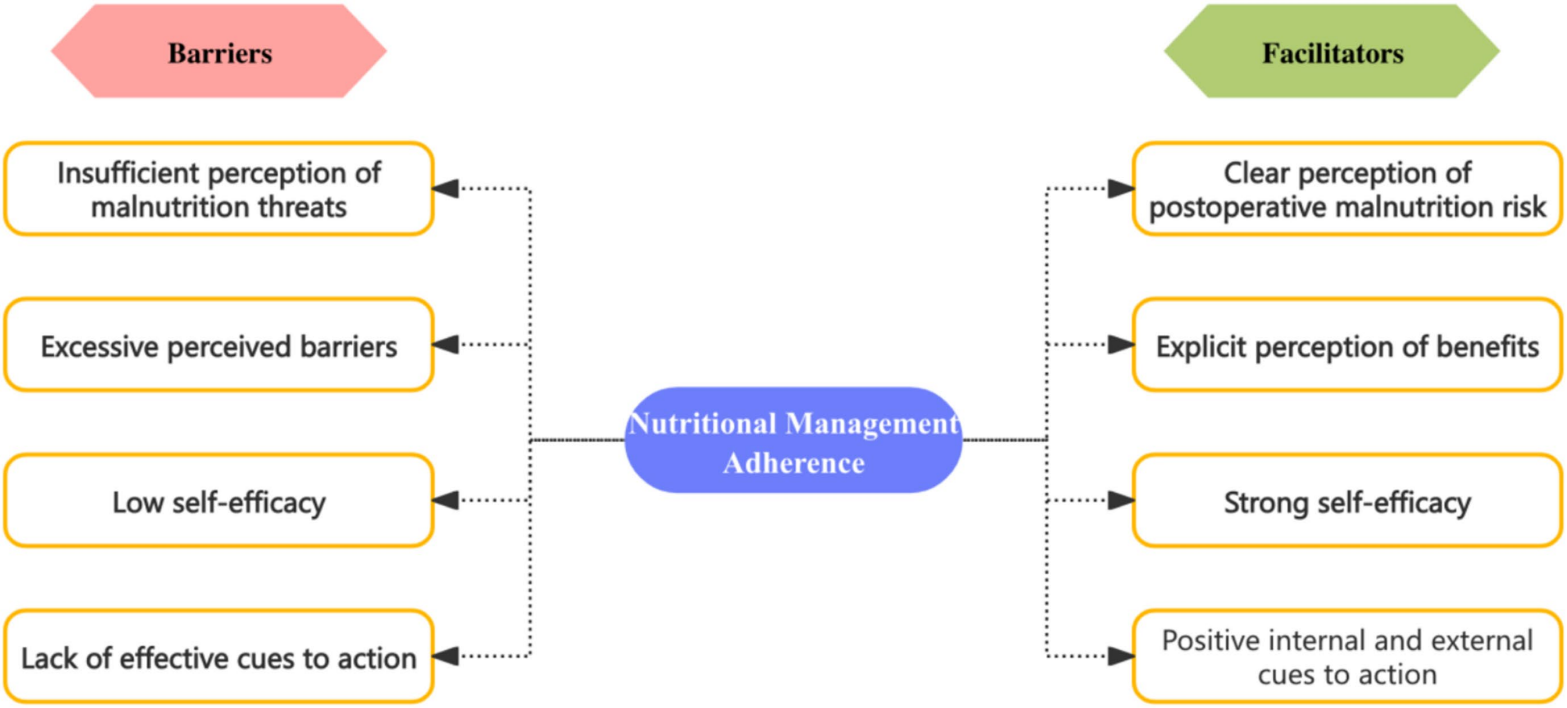
Influencing factors of nutritional management adherence in patients after esophageal cancer surgery.

A total of 18 participants were interviewed. Table 1 shows the characteristics of the participants. From our analysis, guided by the HBM theory, finally, the barriers and facilitators influencing adherence to home-based nutritional management in patients after esophageal cancer surgery were identified, as shown in Figure 2 and Table 2.

**Table 1:** Participant characteristics (*n* = 18).

ID	Age	Sex	Education level	Nutritional support route	Time after surgery
P1	65	Male	Junior high school	Oral	3 months
P2	67	Female	Primary school	Oral	1 month
P3	55	Female	High school	Oral	2 month
P4	59	Male	Primary school	Enteral + oral	1 month
P5	54	Male	High school	Oral	6 month
P6	66	Female	Primary school	Oral	4 month
P7	59	Female	Junior high school	Enteral + oral	1 month
P8	62	Male	Primary school	Oral	7 month
P9	60	Male	Junior high school	Oral	5 month
P10	55	Male	High school	Enteral + oral	2 month
P11	59	Male	Junior high school	Enteral + oral	1 month
P12	70	Female	Primary school	Oral	3 month
P13	67	Male	Primary school	Oral	2 month
P14	73	Male	Primary school	Oral	3 month
P15	57	Male	Junior high school	Oral	2 month
P16	64	Male	Junior high school	Enteral + oral	1 month
P17	50	Male	Bachelor’s degree	Oral	8 month
P18	58	Male	High school	Oral	3 month

**Table 2:** Identified barriers and facilitators of home-based nutritional management adherence in patients after esophageal cancer surgery according to HBM.

Factors	HBM	Results
Barriers	Perceived susceptibility Perceived severity	Insufficient perception of malnutrition threats Lack of awareness of long-term malnutrition risks Cognitive biases about nutritional management
	Perceived barriers	Excessive perceived barriers Symptom burden interfering with eating Financial burden and negative emotions Lack of nutrition knowledge and skills
	Self-efficacy	Low self-efficacy
	Cues to action	Lack of effective cues to action
Facilitators	Perceived susceptibility Perceived severity	Clear perception of postoperative malnutrition risk
	Perceived benefits	Explicit perception of benefits
	Self-efficacy	Strong self-efficacy Active learning and strategy development Positive feedback loop from experience to confidence Internalization of nutritional management
	Cues to action	Positive internal and external cues to action Clear internal cues: sensitization to physical signals and self-monitoring Strong external cues: synergistic effects of multi-source support networks

### 3.1 Barriers

#### 3.1.1 Insufficient perception of malnutrition threats

##### 3.1.1.1 Lack of awareness of long-term malnutrition risks

Due to a lack of knowledge, some patients were found to focus only on short-term, immediate discomfort, such as physical weakness or fatigue, failing to recognize the long-term, severe, and irreversible health threats of malnutrition. Moreover, they did not connect home nutritional management with long-term recovery outcomes or survival risks. This “cognitive blind spot” significantly weakened their intrinsic motivation to manage nutrition well. Some patients even attributed the core manifestations of malnutrition, such as persistent weight loss and fatigue, to unavoidable factors like “tumor consumption” or “surgical trauma.”

*P3: “I just eat less now. No other discomfort. Sometimes I get short of breath if I walk too much, but I feel I’m recovering well.”*

*P6: “I think it’s fine as long as I can eat. My weight is a bit down, but it will gradually improve.”*

*P11: “My illness affects eating anyway. It’s normal to be thinner now. I don’t think malnutrition will cause a recurrence.”*

##### 3.1.1.2 Cognitive biases about nutritional management

Some patients have oversimplified systematic nutritional management as supplementing with a few nutrients, believing that eating more meat, eggs, and milk would solve this problem. They failed to understand the importance of comprehensive and balanced nutrition for tissue repair and the maintenance of body function.

*P2: “I eat three or four meals a day, each a big bowl. My legs feel strong. I eat eggs and meat every day. I think my nutrition is adequate.”*

*P8: “Last time the doctor said my protein was low and my nutrition was insufficient. After coming home, I eat an extra egg every day. That should be enough.”*

*P13: “My family told me bone broth is nutritious, so I drink two big bowls every day—it’s better than anything. Those nutrition powders are pricey and taste bad. No point drinking them.”*

#### 3.1.2 Excessive perceived barriers

##### 3.1.2.1 Symptom burden interfering with eating

The patient experienced dysphagia, reflux, diarrhea, and early satiety. These symptoms directly affect the physiological experience and psychological willingness to eat, making them “afraid to eat,” and thereby hindering adherence to nutritional guidance.

*P1: “I have a good appetite and want to eat more, but sometimes one extra bite causes stomach pain—once it hurt so much I sweated profusely and took a long time to recover. Now I’m afraid to eat.”*

*P5: “My reflux is severe. As soon as I lie down, acid rises to my throat, burning my chest. I don’t dare eat fruit, and I especially avoid dinner.”*

*P10: “I feel full after just a few bites, my stomach bloats like a drum, and I feel I’ll vomit if I eat another bite. I know I haven’t eaten enough, but I really can’t.”*

##### 3.1.2.2 Financial burden and negative emotions

Beyond physical symptoms, financial pressure weakens the patients’ ability and willingness to follow nutritional plans.

*P5: “I used to take care of my family; now they have to revolve around me. I know they are stressed too—I am a burden.”*

*P9: “We’ve spent so much money on treatment, and I can’t earn anything now. Since I can’t eat much anyway, I just eat with the family—no special preparation.”* In addition, boredom caused by dietary monotony, social isolation due to not being able to eat with others, and stigma constitute psychological barriers.

*P14: “Eating the same things every day, I lose my appetite. Before, the family ate happily together; now I sit aside eating some paste—it’s really upsetting.”*

*P15: “Since I learned about my illness, I just want to be alone. I don’t want to talk much with family or friends, and I’m afraid to hear their comfort. I’m not interested in anything, don’t want to eat, and don’t want to bother preparing food.”*

##### 3.1.2.3 Lack of nutrition knowledge and skills

Patients and caregivers were found to lack knowledge regarding food selection, preparation, and tube maintenance.

*P2: “The nurse said to eat high-protein foods, but besides meat, eggs, and milk, what else? I don’t know much about that.”*

*P4: “My family wants to boost my nutrition and often makes soups. I’ve heard that herbs like ginseng and astragalus are good for the body, but we dare not add them arbitrarily—we don’t know the right combinations.”*

*P7: “In the hospital, nurses did everything. We thought it looked easy. But when we had to do it ourselves—how fast to infuse, how to flush the tube—we were afraid of making mistakes. Every step made us anxious.”*

*P16: “The tube makes my nose red and sore, and no matter how I tape it, it’s uncomfortable. We don’t know how to adjust it.”*

#### 3.1.3 Low self-efficacy

Patients generally had low self-efficacy in home nutritional management. They also severely lacked confidence in their ability to overcome difficulties as well as successfully implement and adhere to the nutritional plans. This collapse of intrinsic beliefs was found to be a key psychological barrier to maintaining healthy behaviors. This manifested as frustration, dependence, and helplessness.

*P1: “My family follows the recipes strictly, even consulting doctors and nurses, but I still have reflux. I’ve lost confidence. Maybe my body is just like this—no matter what I do, it won’t help.”*

*P4: “I rely entirely on my wife for food. She decides what to eat. I don’t know how to do it, and I’m afraid I’ll make things worse if I try.”*

*P12: “I’m already this ill. Just surviving is enough. Nothing I eat matters anymore.”*

#### 3.1.4 Lack of effective cues to action

Patients lacked continuous, personalized, and trustworthy guidance and reminders, making it difficult for them to initiate and maintain nutritional behaviors.

*P5: “When I was discharged, the doctor kept saying ‘strengthen nutrition,’ but how exactly? How much should I eat each day? What should I do if I feel uncomfortable after eating? The doctor didn’t tell us.”*

*P8: “Before discharge, the doctor and nurse explained how to eat, but when I got home, I had no idea whether I was doing it right or if I was getting enough nutrition.”*

*P13: “One person says this is good, another says that is more nutritious. The more I listen, the more confused I get. I wish a professional could organize this information for me.”*

### 3.2 Facilitators

#### 3.2.1 Clear perception of postoperative malnutrition risk

Unlike patients with insufficient threat perception, some had a clear, accurate, and strong awareness of the risk of postoperative malnutrition, which directly served as a warning. This transformation of abstract risk into an imminent, severe, and personalized threat was a key driver for adopting and persisting with nutritional management behaviors.

*P8: “My weight hasn’t increased—it’s even dropped a bit. I don’t think that’s a good sign. I checked: if weight keeps dropping after surgery, immunity collapses, and infections or other complications may occur. So I came for a check-up immediately.”*

*P9: “After returning home, I was very worried about not eating well. Now I eat to save my life. Only with good nutrition can I recover.”*

*P16: “The doctor said that for us, eating well and maintaining nutrition is the foundation—only then can my body tolerate subsequent chemotherapy.”*

#### 3.2.2 Explicit perception of benefits

When patients clearly perceived the specific and positive health outcomes of following the nutritional regimen, this perceived benefit became the most direct and powerful intrinsic motivation for maintaining healthy behaviors. Tangible physical improvements were found to create strong positive feedback loops.

*P6: “At this follow-up, my albumin level improved, and I gained one kilogram compared to last month. Although eating is still difficult, seeing the numbers on the scale and gradually regaining strength makes me feel that all the daily effort is worthwhile.”*

*P18: “Following your advice—small, frequent meals and walking half an hour after eating—my reflux and heartburn have really decreased. I feel I’ve found the right method, so I’m more confident to continue.”*

Such “effort pays off” experiences directly reinforced their behavior.

#### 3.2.3 Strong self-efficacy

In contrast to those with low self-efficacy, some patients developed strong self-efficacy through active cognitive restructuring and behavioral practices. This firm belief in their ability to successfully manage home nutrition became the core intrinsic driver for overcoming barriers and maintaining healthy behaviors. This was characterized by a shift from passive acceptance to active exploration and from dependence on others to self-mastery.

##### 3.2.3.1 Active learning and strategy development

When faced with eating difficulties, patients with strong self-efficacy showed strong learning motivation and problem-solving orientation. They actively sought information and developed personalized dietary strategies.

*P10: “The doctor said to eat high-protein foods, so I searched online and watched videos. I finally understood which foods are high-quality proteins. During follow-up visits, I often exchange experiences with other patients and learn soft-food combinations and preparation methods.”*

*P17: “I found that small, frequent meals didn’t suit me—they caused reflux. So I experimented and returned to three main meals a day, walking slowly after each meal. My reflux improved a lot. You have to find what works for you.”*

##### 3.2.3.2 Positive feedback loop from experience to confidence

Every small success in home nutritional management became a driver for improving self-efficacy, forming a virtuous cycle of “success → confidence → further success.”

*P13: “At first I was even afraid to drink water. Then I gradually realized that as long as I didn’t eat too fast or take too big bites, it was fine. This gave me more confidence in eating.”*

*P18: “Now I know very clearly what to eat and how to eat. If a meal makes me uncomfortable, I can analyze whether it was the wrong food or eating too fast, and then adjust.”*

##### 3.2.3.3 Internalization of nutritional management

Strong self-efficacy eventually led patients to internalize professional nutritional requirements as part of their personal health responsibility, transforming nutritional management from a “task” into a “commitment.”

*P3: “Now I watch videos every day about post-cancer nutrition and learn to cook those dishes myself. I’m not eating for others, so it’s not a task—it’s for myself.”*

*P7: “Only with good nutrition can I recover, so I pay great attention to what I eat every day.”*

*P17: “I used to just eat to fill my stomach, but I don’t see it that way now. I know that what I eat matters for my recovery, so I check out which foods help with recovery and which I should avoid. Nobody’s forcing me, I just want to do this.”*

#### 3.2.4 Positive internal and external cues to action

Cues to action are the key triggers that transform health beliefs into actual behaviors. A set of positive, diverse, and sustained internal and external cues together were found to constitute an effective behavioral “nudge system” that could effectively trigger, guide, and maintain home-based nutritional management behaviors.

##### 3.2.4.1 Clear internal cues: sensitization to physical signals and self-monitoring

Patients learned to interpret perceived bodily changes as positive prompts to take action, shifting from passive sensation to active interpretation.

*P2: “Previously I thought fatigue was just due to illness. Now when I feel weak, I wonder, ‘Did I get enough protein today?’ and then I drink some milk or a protein supplement.”*

*P9: “We bought a smart scale and weigh at the same time every day. It generates a trend curve, and I use that curve to guide my family in adjusting my recipes.”*

##### 3.2.4.2 Strong external cues: synergistic effects of multi-source support networks

Effective external prompts from the healthcare system, family, and peers provided continuous behavioral navigation and motivation.

*P1: “The WeChat group the nurse added me to before discharge is very useful. I can ask questions directly and feel reassured.”*

*P6: “My family turned my eating into a check-in activity, with my little grandson supervising me. Completing it well earns a reward. This makes eating feel less like a solitary struggle and easier to sustain.”*

*P10: “I added a fellow patient on WeChat. He has a great attitude and recovers well. We often exchange ideas about diet and exercise—he has had a big influence on me.”*

## 4 Discussion

This study adopts the HBM as its core theoretical framework to interpret empirical findings. Facilitators and barriers derived from interview data were systematically assigned to the fundamental constructs of the model. Focusing on the key research issue of insufficient long-term adherence to home-based nutritional management, the discussion section is organized into three interrelated thematic domains: cognitive restructuring, barrier screening and targeted mitigation, and multi-stakeholder synergistic empowerment. Such thematic structuring enables a comprehensive dissection of the mechanistic pathways via which each determinant modulates patients’ adherence behaviors, while further unpacking the theoretical connotations of the present findings.

### 4.1 Reshaping patients’ perception of nutritional risk and benefits to improve adherence

Our findings show that insufficient threat perception and cognitive biases are key barriers to home-based nutritional management, consistent with the findings of Li et al. (16). Research has shown that cancer patients generally have low levels of nutritional knowledge, and up to 99.6% have dietary misconceptions (17, 18). Patients often fail to recognize the long-term risks of malnutrition, attributing postoperative weight loss to the natural course of the disease or simplifying nutritional management to simply eating more meat, eggs, and milk. They do not establish causal links between malnutrition and serious outcomes, such as infection or recurrence.

Therefore, when providing nutritional education, healthcare providers should avoid vague statements such as “strengthen nutrition—it is good for you.” Instead, they should use patients’ own experiences (e.g., persistent weight loss and fatigue) or invite them to share their stories, directly linking observable signs of malnutrition (e.g., continued weight loss and weakness) with abstract long-term consequences (e.g., increased infection risk and reduced chemotherapy tolerance). This transforms nutritional risk from a vague concept into a tangible, specific, personal threat. At the same time, guide patients to use visual tools, such as weight change curves and laboratory trend charts, to observe their progress, thereby reinforcing the driving role of health beliefs in nutritional management behaviors.

Furthermore, inpatient nutritional education alone is insufficient to ensure post-discharge adherence. This study identified fragmented health education in current post-esophagectomy care, where instruction typically terminates at discharge, leaving patients in an information vacuum and exacerbating non-adherence. Accordingly, early discharge planning, a transitional care model initiated at admission, maintained throughout hospitalization, and extended post-discharge, is recommended. Through structured education, written materials, and remote follow-up, this model transforms health education from one-time instruction into continuous empowerment, ultimately enhancing home-based nutritional adherence. These findings support incorporating early discharge planning into standard perioperative nursing protocols for esophageal cancer, with corresponding quality assurance measures.

### 4.2 Precisely identify and address multiple execution barriers and build a multi-dimensional support system

Our study found that patients face multiple execution barriers during home-based nutritional management, including physical symptom burden, negative psychological emotions, and knowledge/skill deficits, all of which affect their confidence in participating in nutritional management. Research has shown that postoperative patients with esophageal cancer commonly experience nutrition-impacting symptoms, often with multiple coexisting symptoms (19, 20)^.^ Symptom management is a prerequisite to ensure adequate nutrition. For patients with reflux, bloating, nausea, or vomiting, advise dietary adjustments (small, frequent meals; avoiding gas-producing foods); use traditional Chinese medicine alternative therapies such as acupoint application (21), press-needles (22), Chinese herbal hot packs (23), or auricular acupressure (24); when necessary, prokinetic medications may be used to minimize symptom interference with eating.

Feelings of guilt due to financial burden, anxiety, fear of symptoms, and loneliness due to social isolation strongly affect patients’ willingness to engage in home-based nutritional management. The tendency to “settle for whatever the family eats” because of a perceived burden is particularly noteworthy. This indicates that nutritional management is not merely a physiological issue but also involves family emotions and support.

Therefore, nutritional interventions should not be limited to knowledge and skill transfer; psychological and family support must be incorporated. During follow-up, clinicians must actively assess patients’ emotional status; use open-ended questions to encourage them to express their inner struggles; apply psychological support techniques such as mindfulness-based stress reduction, relaxation training, and cognitive restructuring, to alleviate negative emotions; guide family members to provide companionship, listening, and encouragement in appropriate ways; and help families face financial problems constructively.

Lack of nutrition knowledge and skills is a major cause of practical difficulties, with “vague quantification standards” and “lack of actionable recipes” being two prominent issues. To address this issue, we recommend developing visual standardized nutrition education tools that translate abstract protein and calorie requirements into understandable and actionable daily food portions. Based on the patients’ dietary needs at different stages, specific recipes and a related app that embeds these recipes should be developed as examples, allowing patients to perform equivalent food substitutions or customize their own recipes.

### 4.3 Strengthen the synergistic effect of self-efficacy and cues to action to improve adherence

Low self-efficacy and a lack of cues to action have been found to interact with and reinforce each other, jointly restricting adherence to home-based nutritional management after esophageal cancer surgery (25)^.^ Self-efficacy is the internal engine that transforms cues into action. Patients with high self-efficacy actively interpret cues as signals and respond positively to the same. Cues to action serve as a bridge translating self-efficacy into actual behavior; continuous, clear cues help patients start acting even when confidence is not yet fully established, and subsequent success experiences further boost self-efficacy (26).

Therefore, when designing interventions to improve adherence to nutritional management, the synergistic effect of these two factors should be fully considered, and a phased plan should be implemented.

Discharge preparation phase: Assessing patient self-efficacy. For those with low self-efficacy, the frequency and intensity of external support should be increased; for example, proactive follow-up calls or video calls on days 3, 7, and 14 post-discharge to check nutritional implementation and provide immediate feedback. A “daily care checklist” specifying meal reminders, weight measurement, symptom observation, and other tasks should be developed.

Behavior maintenance phase: Gradually shifting cues from external dependence to internalization. Guiding patients and caregivers to maintain a “diet diary” that records daily food types, amounts, and physical responses. Setting phone alarms for weighing and plotting weight change curves.

Setback phase: Establish alert indicators (e.g., >1 kg weight loss in a week, severe adverse reactions) to trigger urgent follow-ups with the nurse.

## 5 Limitations

This study had several limitations. First, it was conducted at a single center (a tertiary hospital in Henan Province), which limits the generalizability of our findings to other regions or primary care settings. Second, the sample size was relatively small (*n* = 18) and the study did not capture the diverse experiences of patients across different disease stages or nutritional support routes. Third, retrospective interviews may have introduced a recall bias, particularly among patients with longer post-discharge intervals. Fourth, as this was an exploratory qualitative study, we did not quantify the relative weight of each influencing factor. Future research needs to employ multi-center, large-sample mixed-methods designs as well as incorporate structural equation modeling and longitudinal follow-up. This would enable researchers to capture the dynamic trajectories of patients’ health beliefs and adherence behaviors and provide evidence for developing staged, precision-based transitional nutritional care programs.

## 6 Conclusion

Guided by the HBM, this study systematically explored the barriers and facilitators of adherence to home-based nutritional management among patients after esophageal cancer surgery, validated the applicability of this theory in the field of oncology rehabilitation nursing, and identified modifiable behavioral targets. In clinical practice, a closed-loop management process of “assessment-identification-intervention-feedback” should be established, integrating structured education, teach-back methods, and individualized goal-setting throughout the patient’s hospital stay. Furthermore, early discharge planning should be incorporated into the standard perioperative nursing care plan for esophageal cancer, with multidisciplinary teams collaboratively developing a smooth transition plan from hospital to home. This approach bridges structured education with post-discharge follow-up, fills the post-discharge vacuum in information and actionable cues, translates professional nutritional advice into sustainable daily practices, and ultimately improves the long-term nutritional status and quality of life of patients.

## Data Availability

The datasets presented in this article are not readily available due to ethical and privacy restrictions. Requests to access the datasets should be directed to the corresponding author.
